# Draft Genome Sequence of *Escherichia coli* O157:H7 ATCC 35150 and a Nalidixic Acid-Resistant Mutant Derivative

**DOI:** 10.1128/genomeA.00734-15

**Published:** 2015-07-23

**Authors:** James A. Markell, Adam G. Koziol, Dominic Lambert

**Affiliations:** a Canadian Food Inspection Agency, Ottawa, Ontario, Canada; b Carleton University, Ottawa, Ontario, Canada

## Abstract

Shiga toxin-producing *Escherichia coli* strains, occasionally isolated from food, are of public health importance. Here, we report on the 5.30-Mbp draft genome sequence of *E. coli* O157:H7 EDL931 (strain ATCC 35150) and the 5.32-Mbp draft genome sequence of a nalidixic acid-resistant mutant derivative used as a distinguishable control strain in food-testing laboratories.

## GENOME ANNOUNCEMENT

EDL931 (ATCC 35150) is an *Escherichia coli* O157:H7 strain first isolated from fecal samples from patients suffering from an outbreak of gastrointestinal illness in Oregon and Michigan in 1982. It was strongly suspected to be the cause of the illness and was later confirmed to be pathogenic by Beery et al in 1984 (1, 2). A nalidixic acid-resistant mutant strain (Nal^r^) of EDL931 was produced to be used as a positive-control strain in the food-testing laboratories of the Canadian Food Inspection Agency (3).

Genomic DNA was isolated from overnight cultures of the parent and Nal^r^ mutant strain grown in nutrient broth using the Promega Maxwell 16 cell DNA purification kit (Promega, Madison, WI). Sequencing libraries were constructed using the Nextera XT DNA sample preparation kit, and paired-end sequencing was performed on the MiSeq platform (Illumina, Inc., San Diego, CA) using a 500-cycle MiSeq reagent kit (version 2). A total of 959,784 and 1,746,204 paired-end reads were generated for EDL931 and the Nal^r^ mutant, respectively. Sequencing errors in the reads were corrected using Quake version 0.3, with a *k*-mer size of 15 (4). The genomes were assembled *de novo* using SPAdes version 3.1.1 (5). Contigs <1,000 bp were excluded from the analysis. Assembly of the EDL931 reads resulted in 242 contigs, with an average 31-fold coverage. The combined length of the draft genome is 5.30 Mbp, with a G+C content of 50.42%. Assembly of the mutant strain reads resulted in 143 contigs (all contigs <1,000 bp were eliminated), with an average 53-fold coverage. The combined length of the mutant draft genome is 5.32 Mbp, with a G+C content of 50.28%. Gene predictions and annotations were performed with the National Center for Biotechnology Information (NCBI) Prokaryotic Genome Annotation Pipeline (PGAP) (6), which predicted 5,271 coding sequences (CDSs) and 13 rRNA, 88 tRNA, and 15 noncoding RNA (ncRNA) genes for EDL931. A total of 5,286 CDSs and 13 rRNA, 90 tRNA, and 15 ncRNA genes were predicted for the Nal^r^ strain. This is compared to 5,149 CDSs and 5 rRNA, 86 tRNA, and 19 ncRNA genes predicted in a previously published *E. coli* O157:H7 strain EDL931 genome (GenBank accession no. AWXM00000000.2). The nalidixic acid-resistant mutant strain showed a single nucleotide difference compared to the parental strain, as determined using kSNP version 2 with a *k*-mer size of 51 (7). The base change resulted in a serine-to-asparagine mutation at position 83 within the *gyrA* gene, consistent with previous studies on the development of quinolone resistance in *E. coli* (8).

### Nucleotide sequence accession numbers.

These whole-genome shotgun projects have been deposited at DDBJ/EMBL/GenBank under accession numbers JXUS00000000 and JYIO00000000. The versions described are JXUS00000000.1 and JYIO00000000.1, respectively.

## References

[1] Riley LW, Remis RS, Helgerson SD, McGee HB, Wells JG, Davis BR, Hebert RJ, Olcott ES, Johnson LM, Hargrett NT, Blake PA, Cohen ML 1983 Hemorrhagic colitis associated with a rare *Escherichia coli* serotype. N Engl J Med 308:681–685. doi:10.1056/NEJM198303243081203. 6338386

[2] Beery JT, Doyle MP, Higley NA 1984 Cytotoxic activity of *Escherichia coli* O157:H7 culture filtrate on the mouse colon and kidney. Curr Microbiol 11:335–342. doi:10.1007/BF01567702.

[3] Blais BW, Martinez-Perez A, Gauthier M, Allain R, Pagotto F, Tyler K 2008 Development of unique bacterial strains for use as positive controls in the food microbiology testing laboratory. J Food Prot 71:2301–2306. 19044277 10.4315/0362-028x-71.11.2301

[4] Kelley DR, Schatz MC, Salzberg SL 2010 Quake: quality-aware detection and correction of sequencing errors. Genome Biol 11:R116. doi:10.1186/gb-2010-11-11-r116. 21114842 PMC3156955

[5] Bankevich A, Nurk S, Antipov D, Gurevich AA, Dvorkin M, Kulikov AS, Lesin VM, Nikolenko SI, Pham S, Prjibelski AD, Pyshkin AV, Sirotkin AV, Vyahhi N, Tesler G, Alekseyev MA, Pevzner PA 2012 SPAdes: a new genome assembly algorithm and its applications to single-cell sequencing. J Comput Biol 19:455–477. doi:10.1089/cmb.2012.0021. 22506599 PMC3342519

[6] Tatusova T, DiCuccio M, Badretdin A, Chetvernin V, Ciufo S, Li W 2013 Prokaryotic Genome Annotation Pipeline. *In* The NCBI handbook, 2nd ed. National Center for Biotechnology Information, Bethesda, MD. http://www.ncbi.nlm.nih.gov/books/NBK174280/.

[7] Gardner SN, Hall BG 2013 When whole-genome alignments just won’t work: kSNP v2 software for alignment-free SNP discovery and phylogenetics of hundreds of microbial genomes. PLoS One 8:e81760. doi:10.1371/journal.pone.0081760. 24349125 PMC3857212

[8] Sáenz Y, Zarazaga M, Briñas L, Ruiz-Larrea F, Torres C 2003 Mutations in *gyrA* and *parC* genes in nalidixic acid-resistant *Escherichia coli* strains from food products, humans and animals. J Antimicrob Chemother 51:1001–1005. doi:10.1093/jac/dkg168. 12654733

